# Adaption of an Episomal Antisense Silencing Approach for Investigation of the Phenotype Switch of *Staphylococcus aureus* Small-Colony Variants

**DOI:** 10.3389/fmicb.2019.02044

**Published:** 2019-09-04

**Authors:** Nina Schleimer, Ursula Kaspar, Britta Ballhausen, Sarah A. Fotiadis, Jessica M. Streu, André Kriegeskorte, Richard A. Proctor, Karsten Becker

**Affiliations:** ^1^Institute of Medical Microbiology, University Hospital Münster, Münster, Germany; ^2^Departments of Medical Microbiology/Immunology and Medicine, School of Medicine and Public Health, University of Wisconsin, Madison, WI, United States

**Keywords:** *Staphylococcus aureus*, small-colony variant (SCV), antisense silencing, phenotype switch, enoyl-acyl-carrier-protein reductase gene (*fabI*), *tet*-regulatory expression system

## Abstract

*Staphylococcus aureus* small-colony variants (SCVs) are associated with chronic, persistent, and relapsing courses of infection and are characterized by slow growth combined with other phenotypic and molecular traits. Although certain mechanisms have been described, the genetic basis of clinical SCVs remains often unknown. Hence, we adapted an episomal tool for rapid identification and investigation of putative SCV phenotype-associated genes via antisense gene silencing based on previously described Tn*l0*-encoded *tet*-regulatory elements. Targeting the SCV phenotype-inducing enoyl-acyl-carrier-protein reductase gene (*fabI*), plasmid pSN1-AS‘*fabI*’ was generated leading to antisense silencing, which was proven by pronounced growth retardation in liquid cultures, phenotype switch on solid medium, and 200-fold increase of antisense ‘*fabI*’ expression. A crucial role of TetR repression in effective regulation of the system was demonstrated. Based on the use of anhydrotetracycline as effector, an easy-to-handle one-plasmid setup was set that may be applicable to different *S. aureus* backgrounds and cell culture studies. However, selection of the appropriate antisense fragment of the target gene remains a critical factor for effectiveness of silencing. This inducible gene expression system may help to identify SCV phenotype-inducing genes, which is prerequisite for the development of new antistaphylococcal agents and future alternative strategies to improve treatment of therapy-refractory SCV-related infections by iatrogenically induced phenotypic switch. Moreover, it can be used as controllable phenotype switcher to examine important aspects of SCV biology in cell culture as well as *in vivo*.

## Introduction

The small-colony variant (SCV) phenotype represents a slow growing subpopulation of *Staphylococcus aureus* associated with chronic, persistent, and recurring infections, which are particularly difficult to diagnose and challenging in terms of treatment (Proctor et al., 2006; Vaudaux et al., 2006; Kahl et al., 2016). The colony morphology and physiological characteristics of SCVs differ widely from the WT, not only due to slower growth, pinpoint colonies, reduced or no pigmentation and hemolytic activity, respectively, but also in several biochemical traits such as altered expression of virulence factors, decreased respiration and coagulase activity as well as frequently by auxotrophism for hemin, menadione, thymidine, or fatty acids (Proctor et al., 2006; Seggewiß et al., 2006; Kriegeskorte et al., 2011, 2014; Proctor et al., 2014; Schleimer et al., 2018).

The identification of the genetic mechanisms leading to the phenotype switch are prerequisite to prevent SCV-related recurrence and chronic infection and to develop new antimicrobials not selecting for SCVs. Hitherto, however, only some phenotype switch-related genes and mechanisms were identified by *in vitro* generation of knockout mutants (including *accC*, *accD*, *aroD*, *cspB*, *hemA, hemB, menA, menB, menD*, *plsX, sdhCAB*, and *thyA*) and sequencing approaches (including *accC*, *accD*, *aroB*, *aroC*, *aroD*, *ecfA*, *ecfT*, *fabF*, *fabI, hemA, hemB, hemC, hemD, hemE, hemG, hemH, menA, menB, menC, menE, menF, relA*, *stp*, and *thyA*) (von Eiff et al., 1997; Bates et al., 2003; Schaaff et al., 2003; Chatterjee et al., 2008; Lannergård et al., 2008; Duval et al., 2010; Gao et al., 2010; Gaupp et al., 2010; Parsons et al., 2011, 2013, 2014; Köser et al., 2012; Wakeman et al., 2012; Hammer et al., 2013; Dean et al., 2014; Painter et al., 2015; Lin et al., 2016; Cao et al., 2017; Zhang et al., 2017; Bazaid et al., 2018; Giulieri et al., 2018; Schleimer et al., 2018; Vestergaard et al., 2018). Besides these SCVs triggered by mutational events restricted to one gene locus, SCVs being the consequence of combined mutations in two or more genes were also rarely described (Hammer et al., 2013; Bui and Kidd, 2015; James et al., 2019). Moreover, the differential expression of some key transcriptional regulators such as SigB and non-protein-coding RNAs (npcRNAs) were shown to contribute to the phenotype switch (Moisan et al., 2006; Abu-Qatouseh et al., 2010; Mitchell et al., 2013; Bui and Kidd, 2015; Tuchscherr et al., 2015). Nevertheless, the (potential) genetic mechanisms of several clinical SCVs remain unidentified and knockout mutant generation and sequencing approaches including whole genome sequencing (WGS) are time-consuming. Moreover, selective disruption of genes essential for growth is not feasible. Aggravating the analysis of clinical SCVs so far, indubitable identification of genes causative for the phenotype switch is only possible for stable SCVs that already underwent mutational adaption and by comparison with a revertant phenotype that spontaneously emerged from the SCV phenotype (Becker et al., 2006; Schleimer et al., 2018). Besides the rarely isolated stable clinical SCVs, *in vitro* selection for stable SCVs mandatory for WGS as well as for transcriptomics and proteomics can be performed by cultivation in the presence of sublethal concentrations of certain antibiotics (e.g., aminoglycosides) (Balwit et al., 1994) or by serially passaging bacteria in the presence of HeLa cells using modified gentamicin protection assays (McLean et al., 2019). By using these methods, however, the SCV-selective mutations only occur in the corresponding drug target genes or genes essential for antibiotic uptake (Schaaff et al., 2003; McLean et al., 2019). Further approaches selecting for a stable SCV phenotype include a chemostat to generate steady-state growth conditions with low nutrients and low growth rate for a prolonged time and with specific chemical stress (Bui and Kidd, 2015; Bui et al., 2015). Although this is an adequate method, it is also time-consuming and not applicable in every laboratory. Thus, there is an urgent need for simpler, faster, and more cost-effective methods to identify genes that trigger the phenotype switch upon mutational or transcriptional inactivation or downregulation.

**FIGURE 1:**
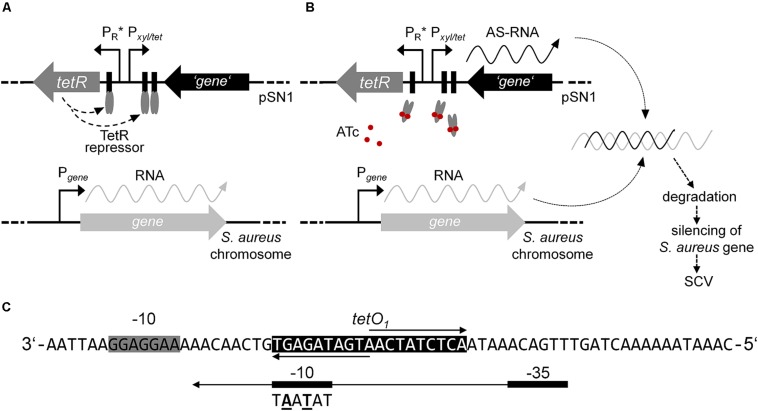
Model of episomal ATc-inducible antisense silencing in *S. aureus* using plasmid pSN1. **(A)** In the absence of effector ATc, constitutively expressed homodimer TetR (gray ellipses) binds to the cognate operator sequences consequently inhibiting gene transcription via promoter P*_*xyl/tet*_* and negatively regulating the rate of transcription of *tetR*. **(B)** Gene expression proceeds upon induction with ATc (red circles) that results in a conformational change of TetR followed by detachment from the cognate operator sequences. Hybridization of transcribed antisense (AS)-RNA to the complementary mRNA is presumptively followed by degradation and silencing of the respective gene maybe resulting in an SCV phenotype. **(C)** Sequence of promoter P_*R*_^∗^ according to Geissendörfer and Hillen (1990); extended Shine-Dalgarno sequence of *tetR* is indicated with a gray box and operator sequence *tetO*_1_ with a black box, respectively. The consensus sequence of the –10 site of promoter P*_*tetR*_* of plasmid pRAB11 is shown below the –10 sequence of P_*R*_^∗^ with the changed bases given in bold and underlined.

Inducible gene expression systems comprising repressor and operator regulatory elements have been reported for use in the genus *Staphylococcus* for selectively controlling the (over)expression of genes (Wieland et al., 1995; Ji et al., 1999; Jana et al., 2000; Helle et al., 2011). Amongst these, tetracycline- (Tc) or anhydrotetracycline (ATc)-inducible expression plasmids comprising improved elements originating from the transposable element Tn*10* were used not only for overexpression, but also for regulated antisense silencing of distinct genes (Ji et al., 1999, 2004; Stary et al., 2010; Helle et al., 2011). This study focused on the adaption of the expression plasmid pRAB11 (Helle et al., 2011) for the identification of genes potentially triggering the phenotype switch from WT to SCV. Therefore, we developed an ATc-inducible antisense approach outlined in Figure 1. This *tet*-regulatory system is based on autoregulated expression of the repressor TetR, which is responsible for the adequate regulation of a target gene. TetR attaches to the cognate operator sequences within both its own promoter and in the divergently located promoter of the target gene, thus, inhibiting the transcription of the target gene (Meier et al., 1988). Upon binding of the effector, TetR undergoes a conformational change and the detachment from the operator proceeds (see Figure 1).

Using the further improved shuttle plasmid pSN1, silencing of the SCV phenotype-related gene *fabI* (encodes for enoyl-acyl-carrier-protein reductase) via production of antisense RNA was performed in a one-plasmid setup. Application of this type of episomal silencing system may provide an easy-to-use and fast tool for the identification of phenotype switch-related genes. The identified genes producing the SCV phenotype will not only allow the screening of clinical SCV isolates, in particular those with undefined auxotrophy, for mutations in these genes by simple Sanger sequencing. Furthermore, knockout mutant generation of the identified genes may also contribute to the identification of possible new auxotrophies and thus to the characterization of undefined SCV types, further simplifying the identification of SCVs. Another advantage of the pSN1 tool is that its inducer ATc is able to penetrate cell membranes. Thus, it may not only help to develop future alternative treatment options of therapy-refractory SCV-related infections, e.g., by iatrogenically induced phenotypic switch, but will also offer the opportunity to monitor the effects and consequences of the switching process on the host cell, organs, and the host organism. Finally, this tool may enable the fast suitability analysis of potential new drug target genes regarding their influence on an adverse phenotype switch to the SCV.

## Materials and Methods

### Bacterial Strains and Plasmids, Growth Conditions, and Antibiotics

All strains and plasmids used are listed in Table 1. *Escherichia coli* cells were cultivated and grown at 37°C in liquid or on solid Luria-Bertani medium (LB; Becton Dickinson, Franklin Lakes, NJ, United States). *S. aureus* strains were grown at 37°C in tryptic soy broth/agar (TSB/TSA; Becton Dickinson) or on Columbia blood agar (BBL^TM^ Columbia agar with 5% sheep blood; Becton Dickinson). All liquid cultures were incubated with shaking at 160 rpm in 10 or 50 mL TSB in 100- or 500-mL glass baffled flasks, respectively. Ampicillin (Sigma-Aldrich, St. Louis, MO, United States) was used at a final concentration of 100 μg/mL for selection of *E. coli.* Chloramphenicol (AppliChem, Darmstadt, Germany) was used at a final concentration of 10 μg/mL for selection of *S. aureus*. ATc (IBA Lifesciences, Göttingen, Germany) was purchased in dry chemical form, prepared as 10 mM stock solution in 70% ethanol and used as effector agent at a final concentration of 0.4 μM.

**TABLE 1:** Bacterial strains and plasmids with their precursors used in this study.

**Strain or plasmid**	**Description**	**Source or references**
*S. aureus* strains		
NCTC8325-4	NCTC8325 derivative; *agr*^+^; 11-bp deletion in *rsbU*; cured of three prophages	Novick, 1967; Herbert et al., 2010
RN4220	NCTC8325-4 derivative; *agr*^–^; 11-bp deletion in *rsbU*; cured of three prophages; MNNG: r^–^m^–^	Iordanescu and Surdeanu, 1976; Herbert et al., 2010
SA113	NCTC8325 derivative; *agr*^–^; 11-bp deletion in *rsbU*; point mutation in *tcaR*; three prophages, Φ11, Φ12, and Φ13; MNNG: r^–^m^–^	Iordanescu and Surdeanu, 1976; Herbert et al., 2010
SAS32/1	SA113 pRAB11	This study
SAS99/1	SA113 pSN1	This study
SAS118/2	SA113 pSN1-AS‘*fabI*’	This study
*E. coli* strain		
One shot^TM^ Top10	*E. coli* K-12 derivative; F- *mcrA* Δ(*mrr-hsd*RMS-*mcr*BC) Φ80*lac*ZΔM15 Δ *lac*X74 *rec*A1 *ara*D139 Δ(*araleu*)7697 *gal*U *gal*K *rps*L (StrR) *end*A1 *nup*G	Invitrogen/Thermo Fisher Scientific
Plasmids		
pCPP-3^1^	*E. coli*/*B. subtilis* shuttle plasmid; derived from plasmids pBR322, pUBll0 and pC194; *neo*	Band et al., 1983
pWH353^1^	*E. coli*/*B. subtilis* shuttle plasmid; pCPP-3 derivative carrying an 800-bp fragment comprising Tn*10* derived elements *tetR* (encoding the TetR repressor) and P_*R*_^∗^ (mutant *tetR* promoter with poly-A block and improved −35 site), and the divergent P*_*xyl/tet*_* fusion promoter from *B. subtilis* with 1x *tetO*; *neo*	Geissendörfer and Hillen, 1990
pSK236^1^	*E. coli*/*Staphylococcus* shuttle plasmid with pUC19 cloned into the *Hin*dIII site of pC194; *bla cat*	(unpublished data) Tinsley and Khan, 2007
pALC2073^1^	*E. coli*/*Staphylococcus* shuttle plasmid; pSK236 derivative with 800-bp fragment from pWH353 comprising *tetR*, P_*R*_^∗^, and P*_*xyl/tet*_* with 1x *tetO* (cloned into the *Pst*I and *Sma*I sites); *bla cat*	Bateman et al., 2001
pRMC2^1^	*E. coli*/*Staphylococcus* shuttle plasmid; pALC2073 derivative with mutated −10 sequence within the *tetR* promoter (5′-tag ag t-3′→ 5′-tat aa t-3′; P*_*tetR*_*) and extended MCS (*Kpn*I, *Hpa*I, *Bgl*II, *Sac*I/*Ban*II, and *Eco*RI); *tetR*, P*_*tetR*_*, and P*_*xyl/tet*_* with1x *tetO; bla cat*	Corrigan and Foster, 2009
pRAB11^2^	*E. coli*/*Staphylococcus* shuttle plasmid; pRMC2 derivative with *tetR*, P*_*tetR*_*, and P*_*xyl/tet*_* with 2x *tetO*; *bla cat*	Helle et al., 2011
pEX-A2	*E. coli* standard plasmid; pUC ori; *bla*	Eurofins Genomics
pEX-A2-P_*R*_^∗^	*E. coli* standard plasmid; pEX-A2 derivative with 90-bp synthetic fragment comprising P_*R*_^∗^ framed by *Xba*I and *Xho*I; *bla*	This study
pSN1	*E. coli*/*Staphylococcus* shuttle plasmid; pRAB11 derivative with *tetR*, P_*R*_^∗^, and P*_*xyl/tet*_* with 2x *tetO*; *bla cat*	This study
pSN1-AS‘*fabI*’	*E. coli*/*Staphylococcus* shuttle plasmid; pSN1 with 382-bp fragment of *fabI* cloned in antisense orientation in the *Eco*RI and *Kpn*I sites downstream of P*_*xyl/tet*_* with 2x *tetO*; *bla cat*	This study

*^1^Precursor plasmids of pRAB11. ^2^Kindly provided by Ralph Bertram, Department of Microbial Genetics, University of Tübingen, Tübingen, Germany.*

### Genetic Manipulation in *E. coli* and *S. aureus*

One Shot^TM^ Top10 chemically competent *E. coli* cells (Invitrogen/Thermo Fisher Scientific, Waltham, MA, United States) were used for propagation and cloning experiments. Transformation of *E. coli* was performed using standard techniques (Hanahan, 1983) and plasmid DNA cloned in *E. coli* was isolated with the Qiagen Plasmid Mini kit (Qiagen, Venlo, Netherlands) and used to transform restriction-deficient *S. aureus* RN4220 by electroporation (Augustin and Götz, 1990). Subsequently, plasmid DNA was introduced into *S. aureus* SA113 by electroporation. All constructed plasmids were verified after each cloning step via standard-PCR amplification using plasmid-specific oligonucleotides pSN1-F (5′-CTGGGCGAGTTTACGGGTTG-3′) and pSN1-R (5′-CAC ATGCAGCTCCCGGAGAC-3′) covering the *tet*-regulatory elements and, if present, the *fabI* fragment. The reaction conditions for standard-PCRs using Taq DNA polymerase (Segenetic, Borken, Germany) were 4 min initial denaturation at 95°C followed by 31 cycles of (i) denaturation at 95°C for 30 s, (ii) annealing at 58°C for 30 s, and (iii) extension at 72°C for 1 min. Final extension was performed at 72°C for 10 min. Analysis of PCR and restriction products was performed by agarose gel electrophoresis and purification using the QIAquick PCR Purification kit (Qiagen). This was followed by Sanger sequencing (Eurofins Genomics, Ebersberg, Germany). Restriction enzymes used for cloning were obtained from New England Biolabs (Frankfurt am Main, Germany). Genomic DNA of NCTC8325-4 was extracted after lysostaphin treatment (20 μg/mL, 1 h, 37°C) (Wak-Chemie Medical, Steinbach, Germany) using the QIAamp DNA Mini kit (Qiagen). Secondary sequence structure prediction was performed using the Mfold web server (Zuker, 2003) with default settings. The structure exhibiting the lowest ΔG was used for further analyses.

### Construction of Plasmids for Episomal Silencing of Gene fabI

To introduce two base substitutions within the −10 site of promoter P*_*tetR*_* in pRAB11, a 90-bp sequence comprising promoter P_*R*_^∗^ (Geissendörfer and Hillen, 1990) framed by *Xba*I and *Xho*I sites was chemically synthesized and cloned into the multiple cloning site (MCS) of pEX-A2 (Eurofins Genomics) to yield pEX-A2-P_*R*_^∗^. Since restriction via *Xba*I and *Xho*I was not practicable, P_*R*_^∗^ was amplified with oligonucleotides P_*R*_^∗^-F (5′-GCGC**TCTAGA**CATCATTAATTCCT-3′) and P_*R*_^∗^-R (5′-CGCG**CTCGAG**GGGATCCAAATAAA-3′) (restriction sites are written in bold) from pEX-A2-P_*R*_^∗^ via standard PCR followed by restriction with *Xba*I and *Xho*I. For the construction of pSN1, the *Xba*I-*Xho*I synthetic P_*R*_^∗^ sequence (see Figure 1C) was cloned into pRAB11 replacing P*_*tetR*_* and generating pSN1 that was transformed to *S. aureus* SA113 to generate SAS99/1.

For the construction of plasmid pSN1-AS‘*fabI*’, a 382-bp fragment of the N-terminal region of the *fabI* gene (SAOUHSC_00947, corresponding to nucleotides 18 to 399) was amplified from genomic DNA of NCTC8325-4 using oligonucleotides *fabI*-AS-F-*Eco*RI (5′-GCGC**GAATTC**CAAAACATATGTCATCATGGGAAT-3′) and *fabI*-AS-R-*Kpn*I (5′-GCGC**GGTACC**TTTAGCTTCATGAG CCACAAT-3′) (restriction sites are written in bold) and the *Eco*RI/*Kpn*I-digested PCR product was ligated in antisense orientation downstream of the P*_*xyl/tet*_* promotor-operator fusion into the *Eco*RI and *Kpn*I sites within the MCS of pSN1. The verified construct was subsequently transformed into SA113 yielding SAS118/2. Further *fabI* fragments with 75-bp, 100-bp, 150-bp, 200-bp, and 300-bp in size originating from different regions of *fabI* with and without the Shine-Dalgarno sequence were additionally amplified (used oligonucleotides and characteristics of each antisense “*fabI*” fragment are listed in Supplementary Tables S1, S2, respectively), cloned into plasmid pSN1, and transformed into SA113.

### Growth Curve Analysis and *tet*-Regulated Antisense Silencing

For growth analysis applying gene silencing due to induction of antisense transcription of the *fabI* fragment, *S. aureus* SA113, SAS32/1, SAS99/1, and SAS118/2 were inoculated in 50 mL TSB (Becton Dickinson) containing chloramphenicol where appropriate. Concurrently, strains were inoculated as stated with and without ATc (IBA). The strains were cultured at 37°C on a rotary shaker at 160 rpm in 500-mL glass baffled flasks. Aliquots were taken every hour to determine the optical density (OD_578__*nm*_).

Furthermore, TSA (Becton Dickinson) containing 0.2 μM and 0.4 μM ATc (IBA) was used for examination of the colony phenotype upon gene silencing. Therefore, samples were adjusted to McFarland 0.5 (in 0.9% NaCl), diluted (10^–4^) and 100 μl were streaked on agar that was then incubated overnight at 37°C. The different colony phenotypes, macroscopically divided into micro-SCVs, mini-SCVs, SCVs, and WTs, were counted and their percentage was calculated (mean of three independent measurements).

### Gene Expression Analysis via Real-Time Quantitative PCR

Levels of *fabI* transcripts at several time points from three independent experiments were determined by real-time qPCR (run in triplicates). Cells were harvested at 2 h, 4 h, and 7 h during growth curve analysis. Harvesting, RNA isolation, DNase treatment, reverse transcription, and qPCR were performed as previously described (Ballhausen et al., 2014) with the exception of minor deviations in the qPCR protocol consisting of 40 cycles of 10 s at 95°C, 10 s at 55°C, and 30 s at 72°C. 16S rRNA (oligonucleotides, 16S-rRNA-1 and 16S-rRNA-2) (Ballhausen et al., 2014) and *gyrB* (*gyrB*-1, 5′-AATTGAAGCAGGCTATGTGT-3′ and *gyrB*-2, 5′-ATAGACCATTTTGGTGTTGG-3′) were used as references. Specific oligonucleotides used for determination of expression of the 382-bp *fabI* fragment were *fabI*-RT-1 (5′-AAATCAACCAGAAGCGCACT-3′) and *fabI*-RT-2 (5′-ACAAGAAGCCTTCACGTGAA-3′) both binding within the antisense fragment. The relative expression was calculated using the software CFX Manager v 3.1 (Bio-Rad Laboratories, Hercules, CA, United States) applying inter-run calibration. Expression values were normalized to strain SAS99/1 (2 h) harboring the empty pSN1 (control) to exclude chromosomal *fabI* expression.

## Results and Discussion

Since SCV-caused infections are difficult to treat, it is of major interest to identify and characterize those genes, which are involved in the phenotypic switch from the WT to the SCV phenotype. However, the approaches used so far for this purpose are expensive, time-consuming, and/or difficult in handling. Moreover, clinically derived strain pairs consisting of both stable SCVs and revertant WTs are prerequisites. Therefore, this study focused on the generation of an easy-to-handle genetic tool for the rapid identification of SCV phenotype generation-related genes.

**FIGURE 2:**
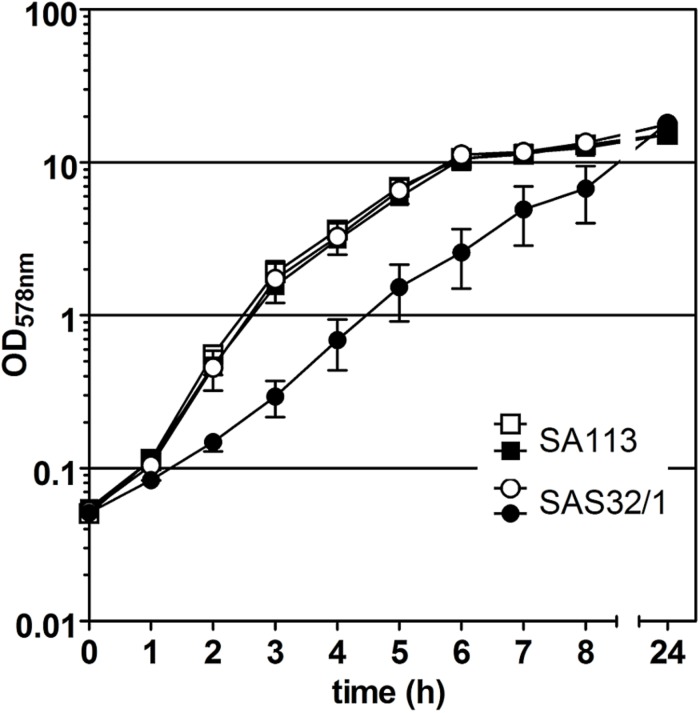
Effect of pRAB11-derived TetR on growth of *S. aureus* strain SAS32/1. *S. aureus* wild type strain SA113 without pRAB11 was used as control. Filled symbols indicate the presence of 0.4 μM anhydrotetracycline (ATc).

**FIGURE 3:**
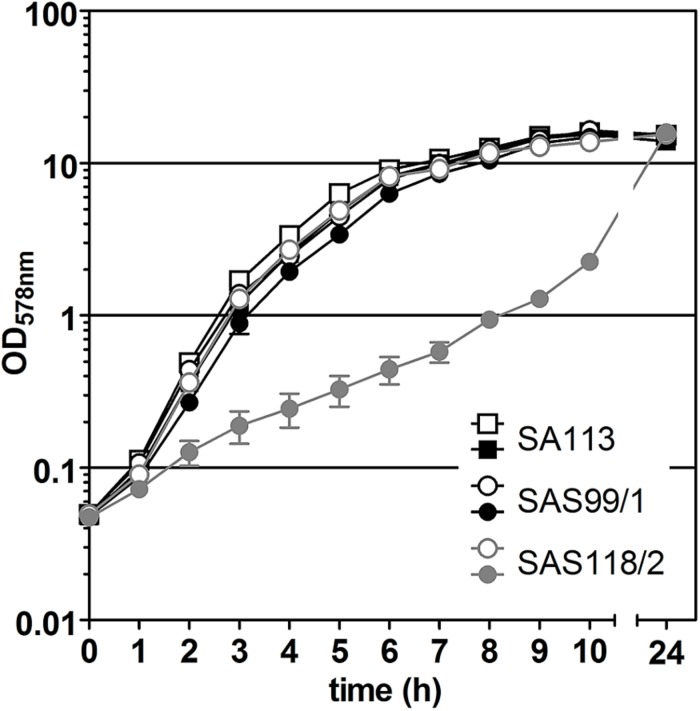
Effect of episomal ‘*fabI*’ antisense expression controlled by pSN1-derived TetR in *S. aureus* SAS118/2 carrying pSN1-AS‘*fabI*’ (gray symbols). Black symbols indicate growth curves of *S. aureus* control strains SA113 and SAS99/1 (SA113 carrying empty pSN1). Filled symbols indicate the presence of 0.4 μM anhydrotetracycline (ATc).

In our study, we adapted the Tn*10*-encoded Tc- or ATc-inducible *tet-*regulated repressor-operator expression system, which was first described in *E. coli* and then modified in several ways to meet the different requirements for Gram-positive bacteria (reviewed in Bertram and Hillen, 2008). For episomal antisense silencing of phenotype-related genes in *S. aureus* (see Figure 1), we employed plasmid pRAB11 (Helle et al., 2011) containing the *tet*-regulatory elements *tetR* (encodes the repressor), its enhanced autoregulated promoter P*_*tetR*_* (Geissendörfer and Hillen, 1990; Corrigan and Foster, 2009), and the divergently located promoter-operator fusion P*_*xyl/tet*_* with two *tetO* sequences (Geissendörfer and Hillen, 1990; see Figure 1). Since for fatty acid metabolism-related gene *fabI*, mutations were already shown to be responsible for the phenotype switch to the SCV (Bazaid et al., 2018), we exemplarily used this gene in our experiments. Initial experiments to determine the suitability of pRAB11 unexpectedly revealed pronounced growth retardation for strain SAS32/1 carrying the empty pRAB11 upon induction with the effector ATc (Figure 2). ATc represents a derivative of Tc, but since growth of SA113 in ATc-containing TSB was not affected, its low antibiotic activity (Degenkolb et al., 1991) was not responsible for the slow growth phenomenon. During generation of plasmid pRAB11 for the use in Gram-positive bacteria, several modifications were performed (see Table 1; Geissendörfer and Hillen, 1990; Bateman et al., 2001; Corrigan and Foster, 2009; Helle et al., 2011). Amongst these, adaptation of the −10 sequence within the *tetR*-driving promoter P_*R*_^∗^ formally designed by Geissendörfer and Hillen (1990) resulted in the consensus sequence (5′-ta**g** a**g** t-3′→ 5′-ta**t** a**a** t-3′) (Corrigan and Foster, 2009), but unintentionally led to a disruption of its palindromic *tetO*_1_ sequence (see Figure 1C) and therefore, presumptively resulted in increased amounts of TetR due to loss of its negative regulation (Stary et al., 2010). Whilst increased amounts of TetR provide improved repression for P*_*xyl/tet*_* promotor constructs harboring only one *tetO* (Corrigan and Foster, 2009) in the uninduced state, these high TetR concentrations may be toxic to the cells (Lutz and Bujard, 1997) as the unbound TetR homodimers are freely available in the induced state. Furthermore, since induction of the P*_*xyl/tet*_* promoter may be negatively affected by overexpression of TetR (Ehrt et al., 2005), we adapted the system by restoring the *tetO*_1_ sequence within the TetR promoter generating plasmid pSN1. *S. aureus* cells harboring pSN1 (SAS99/1) showed no signs of growth retardation in presence of ATc (Figure 3) presumptively linked to the fact that, due to the restored *tetO*_1_, the negatively controlled transcription of *tetR* by TetR as in its original state (Yang et al., 1976; Beck et al., 1982; Wissmann et al., 1988) is reconstituted.

**FIGURE 4:**
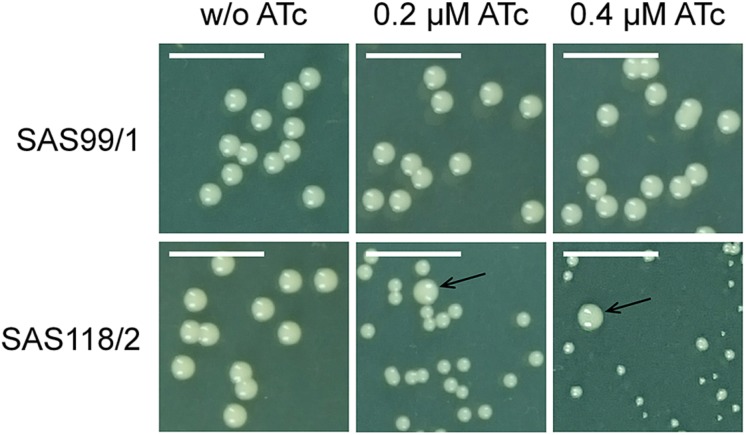
Phenotypic effect of episomal ‘*fabI*’ antisense silencing controlled by pSN1-derived TetR in *S. aureus* on TSA agar with and without anhydrotetracycline (ATc). The upper row shows control strain SAS99/1 harboring the empty plasmid pSN1 and the lower row shows ‘*fabI*’-carrying strain SAS118/2. Phenotype switch from wild type to SCV only occurs if ATc is contained in the medium; arrows indicate unaffected wild type cells. Scale bar indicates 5 mm.

**FIGURE 5:**
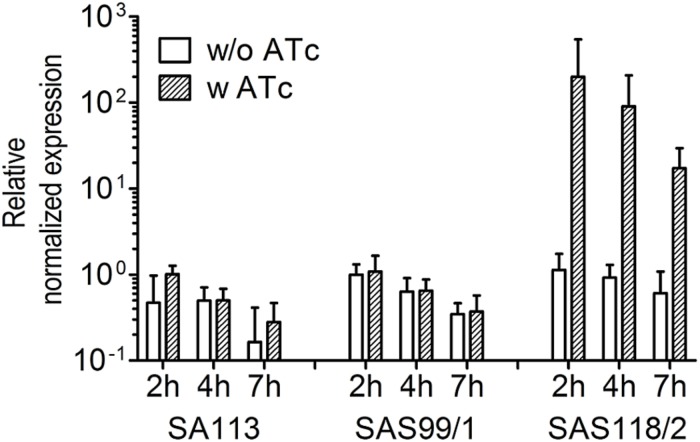
Relative patterns of ‘*fabI*’-antisense expression in *S. aureus* strains SA113, SAS99/1 (carrying pSN1), and SAS118/2 (carrying pSN1-AS‘*fabI*’) in response to 0.4 μM anhydrotetracycline (ATc). Dashed bars indicate the presence of 0.4 μM ATc, the effector of ‘*fabI*’-antisense expression in strains harboring pSN1-AS‘*fabI*’. Normalized expression is shown relative to control SAS99/1 carrying the empty pSN1 (2 h) to exclude chromosomal *fabI* expression.

For episomal antisense silencing, a 382-bp fragment of the gene *fabI* mostly congruent with fragments used before (Ji et al., 2004; Stary et al., 2010) was cloned downstream of promoter P*_*xyl/tet*_* in pSN1. Induction with ATc resulted in distinct retardation of growth (Figure 3) as shown before with chromosomally encoded *tetR* (Stary et al., 2010) as well as for episomal silencing with a plasmid harboring a leaky (Zhang et al., 2000) one-*tetO*-version that was induced with Tc (Ji et al., 2004). Phenotypic characterization of *fabI* antisense silencing on solid agar revealed colonies exhibiting the SCV phenotype only upon induction with ATc (Figure 4). The SCV phenotype was more pronounced on agar containing 0.4 μM ATc than on agar with 0.2 μM ATc. However, on agar with 0.4 μM ATc, SCVs were heterogeneous in size with 74.83% micro-SCVs, 19.14% mini-SCVs, and 3.73% SCVs, respectively. This differentiation into different SCV phenotypes despite the same treatment and environmental conditions is a phenomenon known for SCVs and implicates an adaption strategy during the infection and persistence process (Tuchscherr et al., 2011). It was therefore expected that the bacterial cells have the ability to react slightly different to the silencing of a particular gene and the environmental conditions. Unaffected WT cells remained for both ATc concentrations at a ratio of 1.17% for 0.2 μM and 2.30% for 0.4 μM ATc. This may indicate that some of the cells were able to escape induction with ATc, which needs to be further investigated. Since qPCR showed a considerably elevated expression of antisense ‘*fabI*’ only for strain SAS118/2 in the induced state (∼ 200-fold increase compared to induced control strain SAS99/1, both measured after 2 h of growth) (Figure 5), this indicates functional silencing due to pSN1-derived antisense expression.

Since not all antisense RNA fragments are effective in inhibiting gene expression in *S. aureus* (Ji et al., 2004), we analyzed several *fabI* fragments from different regions of *fabI* with and without Shine-Dalgarno sequence that exhibited 75- to 300-bp in size, a GC content of 29.3 to 36.7% and Δ*G* values of −45.6 to −11.8 kcal/mol (for comparison, the 382-bp fragment exhibited a GC content of 34.8% and a ΔG of −70.3 kcal/mol; see Supplementary Material). However, a silencing effect could only be observed for the 382-bp *fabI* fragment, not for any of the other fragments tested (data not shown) as it could be also not shown before for a fragment bigger than 500 bp (Ji et al., 2004). Therefore, contrary to expectations, the presence of the Shine-Dalgarno sequence does not seem to be relevant as it was described for gene silencing in *E. coli* (Stefan et al., 2008). Secondary sequence structure prediction for each fragment revealed different loop regions with unpaired bases that are complementary and with accessible conformation to hybridize with *fabI* mRNA (data not shown). Even though some of these fragments showed loop regions of a bigger size compared to the 382-bp *fabI* fragment, this seemed to have no impact on silencing. Instead, the quantity of loop regions may contribute to silencing, as the 382-bp *fabI* fragment exhibited 20 loops compared to 4 to 16 loops of the other fragments (see Supplementary Material). Therefore, antisense silencing seems to depend significantly on fragment size and target region. Thus, the final effectiveness of the fragments is difficult to predict necessitating further examinations.

## Conclusion

Our results demonstrate the crucial role of TetR and its major importance in negative regulation of repressor expression for episomal antisense silencing. However, to perform efficient antisense silencing, size and region of the antisense fragment complementary to the region of the target gene remain critical factors that need further detailed investigations. Plasmid pSN1 allowed antisense silencing of the *fabI* gene in an easy-to-handle one-plasmid setup. Due to the MCS directly downstream of the inducible P*_*xyl/tet*_* promoter, genes of interest can easily be cloned into the plasmid. Moreover, the two-*tetO* P*_*xyl/tet*_* version exerted a tight repression of the gene of interest in the uninduced state, thus there is no necessity of chromosomal *tetR* integration prior to use. Also other SCV characteristic (e.g., hemolysis behavior, coagulase and protease production, as well as antibiotic resistance) can be analyzed with this system as already shown for antisense silencing of *hla* (encodes for α-toxin) (Ji et al., 1999). Owing to induction with ATc, pSN1 mediated antisense silencing may not only be applicable to different *S. aureus* backgrounds, but also in cell culture studies (Ji et al., 1999; Bateman et al., 2001). Therefore, pSN1 mediated antisense silencing may not only help to identify SCV phenotype-related genes but also to analyse the consequences of the switching process to the host cells and organs.

## Data Availability

All relevant data for this study are included in the manuscript and/or the Supplementary Files.

## Author Contributions

KB and AK designed the study concept. NS, AK, and BB designed the experiments. NS performed the laboratory work, evaluated the data, and drafted and wrote the manuscript. UK contributed to the data evaluation and writing of the manuscript. SF and JS contributed to the laboratory work. RP provided the scientific support regarding SCVs and interpreted the data. All authors have read and approved the final draft of the manuscript.

## Conflict of Interest Statement

The authors declare that the research was conducted in the absence of any commercial or financial relationships that could be construed as a potential conflict of interest.
