# Supplementary material for: Adaption of an Episomal Antisense Silencing Approach for Investigation of the Phenotype Switch of Staphylococcus aureus Small-Colony Variants
Source: Front Microbiol. 2019 Sep 4;10:2044. doi: 10.3389/fmicb.2019.02044 (PMC6738336; doi:10.3389/fmicb.2019.02044)
Supplement: Supplementary file 1 [file Table_1.pdf]

Supplementary Material

1 Supplementary Tables

Table S1. Oligonucleotides used in this study for amplification of different *fabI* fragments

Oligonucleotide primer[1]	Sequence (5´ → 3´)[2]	Reference
fabI-AS-F-*EcoR*I	GCG C**GA ATT C**CA AAA CAT ATG TCA TCA TGG GAA T	This study
fabI-AS-R-*Kpn*I	GCG C**GG TAC C**TT TAG CTT CAT GAG CCA CAA T	This study
2-AS-*fabI*-F-*EcoR*I	GCG C**GA ATT C**AT TAT AAG GAG TTA TCT TAC ATG TT	This study
2-AS-*fabI*-R-*Kpn*I	GCG C**GG TAC C**AC TTT AGC GAC ACC AAA AGC AAT	This study
3-AS-*fabI*-R-*Kpn*I	GCG C**GG TAC C**TA CGT TCT TTA CGG TAA GTA AAT	This study
4-AS-*fabI*-R-*Kpn*I	GCG C**GG TAC C**TA CGC TTA TTA GCG ATT CCC A	This study
10-AS-*fabI*-R-*Kpn*I	GCG C**GG TAC C**TT GAA TGA TAT ACA CCA TCA ATA TT	This study
11-AS-*fabI*-F-*EcoR*I	GCG C**GA ATT C**GG TTT CAA TAC AAT TCT TAA AGA AA	This study
11-AS-*fabI*-R-*Kpn*I	GCGC**GGTACC**GAATCCGCTATCTACATGAATATT	This study
16-AS-*fabI*-R-*Kpn*I	GCGC**GGTACC**CGCTTCTGGTTGATTTAATTGTTC	This study

1 *fabI*, enoyl-acyl-carrier-protein reductase gene.
2 Restriction sites are given in bold.

Table S2. Characteristics of the antisense *fabI* fragments used for antisense silencing

Oligonucleotide combination[1]	Length (bp)	Region (bp)[2]	GC content (%)	Number of possible structures	Initial ΔG (kcal/mol)[3]	Number of loops[4]
fabI-AS-F-*Eco*RI + *fabI*-AS-R-*Kpn*I	382	+18 to +399	34.8	10	-70.3	20
2-AS-*fabI*-F-*Eco*RI + 4-AS-*fabI*-R-*Kpn*I	75	-20 to +55	29.3	1	-11.8	4
2-AS-*fabI*-F-*Eco*RI + 2-AS-*fabI*-R-*Kpn*I	100	-20 to +80	32.0	2	-12.8	5
2-AS-*fabI*-F-*Eco*RI + 3-AS-*fabI*-R-*Kpn*I	150	-20 to +130	30.7	9	-16.5	8
11-AS-*fabI*-F-*Eco*RI + 11-AS-*fabI*-R-*Kpn*I	150	+607 to +756	36.7	2	-19.0	10
2-AS-*fabI*-F-*Eco*RI + 16-AS-*fabI*-R-*Kpn*I	200	-20 to +180	31.5	6	-30.6	12
2-AS-*fabI*-F-*Eco*RI + 10-AS-*fabI*-R-*Kpn*I	300	-20 to +280	31.3	6	-45.6	16

[1] *fabI*, enoyl-acyl-carrier-protein reductase gene.
[2] Fragments starting at position -20 cover the Shine-Dalgarno sequence.
[3] Secondary sequence structure prediction was performed using the Mfold web server (Zuker, 2003) with default settings and using antisense fragments without restriction sites. The structure exhibiting the lowest ΔG is given.
[4] Amount of loops is given for the structure exhibiting the lowest ΔG.

References

Zuker, M. (2003). Mfold web server for nucleic acid folding and hybridization prediction. *Nucleic Acids Res.* 31, 3406–15. doi:10.1093/NAR/GKG595.
